# FDG-PET/MRI in colorectal cancer care: an updated systematic review

**DOI:** 10.1007/s00261-024-04460-z

**Published:** 2024-07-29

**Authors:** Hui Zhen Lo, Kay Tai Choy, Joseph Cherng Huei Kong

**Affiliations:** 1 School of Medicine, Faculty of Medicine, Nursing and Health Sciences, Monash University, Melbourne, VIC Australia; 2 Department of Surgery, Austin Health, Melbourne, VIC Australia; 3 Department of Surgical Oncology, Peter MacCallum Cancer Centre, Melbourne, VIC Australia; 4 Division of Cancer Research, Peter MacCallum Cancer Centre, Melbourne, VIC Australia; 5 Sir Peter MacCallum Department of Oncology, University of Melbourne, Parkville, VIC Australia; 6 Department of Colorectal Surgery, Alfred Hospital, Melbourne, VIC Australia; 7 Central Clinical School, Monash University, Melbourne, VIC Australia

**Keywords:** FDG-PET/MRI, Colorectal cancer, Accuracy, Standard of care imaging

## Abstract

**Purpose:**

Since its introduction in 2011, FDG-PET/MRI has been advocated as a useful adjunct in colorectal cancer care. However, gaps and limitations in current research remain. This systematic review aims to review the current literature to quantify the utility of FDG-PET/MRI in colorectal cancer care.

**Methods:**

An up-to-date review was performed on the available literature between 2000 and 2023 on PubMed, EMBASE, Medline, databases. All studies reporting on the use of FDG-PET/MRI in colorectal cancer care were analyzed. The main outcome measures were accuracy in initial staging, restaging, and detection of metastatic disease in both rectal as well as colon cancers. The secondary outcome was comparing the performance of FDG-PET/MRI versus Standard of Care Imaging (SCI). Finally, the clinical significance of FDG-PET/MRI was measured in the change in management resulting from imaging findings.

**Results:**

A total of 22 observational studies were included, accounting for 988 patients. When individually compared to current Standard of Care Imaging (SCI)—MRI pelvis for rectal cancer and thoraco-abdominal contrast CT, PET/MRI proved superior in terms of distant metastatic disease detection. This led to as much as 21.0% change in management. However, the technological limitations of PET/MRI were once again highlighted, suggesting SCI should retain its place as first-line imaging.

**Conclusion:**

FDG-PET/MRI appears to be a promising adjunct in staging and restaging of colorectal cancer in carefully selected patients.

**Graphical Abstract:**

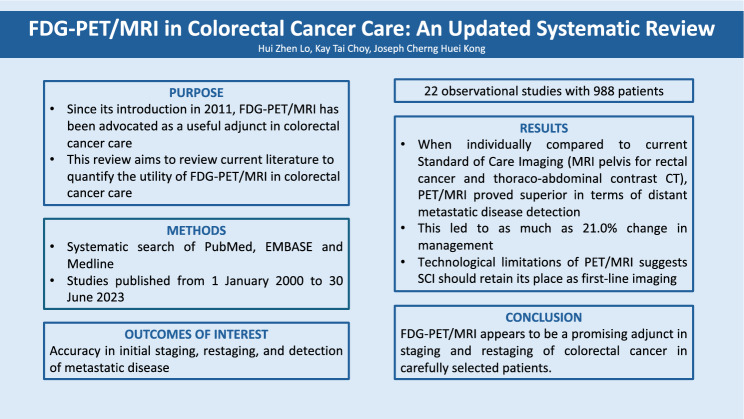

## Introduction

In the management of colorectal cancer, it is vital to obtain an accurate Tumor, Node and Metastasis (TNM) staging to allow for an individualized treatment approach [1, 2]. This aids in complex discussion particularly in locally advanced rectal cancer (LARC) for which clear oncological margins (distal and circumferential resection margin) is paramount. The routine pre-operative work-up (Standard of Care Imaging) for newly diagnosed colorectal cancer patients include computed tomography (CT) to ensure no distant metastasis, magnetic resonance imaging (MRI) for those with mid to low rectal cancers and additionally [^18^F] Fluorodeoxyglucose-positron emission tomography (FDG-PET)/CT if there is concern for any occult residual disease that has not been identified by the first two modalities.

Currently, the treatment algorithm for mid to low rectal cancer is evolving [3]. Historically, any patients with LARC, defined as T3-4 with or without nodal positivity, will either receive neoadjuvant short-course radiotherapy or long-course chemoradiotherapy. However, in the last 2 years, there has been increasing interest in Total Neoadjuvant Therapy (TNT): giving induction (before radiotherapy) or consolidation (after radiotherapy) chemotherapy before definitive curative radical surgery [4, 5]. The basis for this intensified regimen is to bring forward neoadjuvant chemotherapy as patients have higher functional reserves and would better tolerate chemotherapy before major surgery. This would hypothetically reduce the risk of distant recurrence.

Recent intermediate oncological data has shown improvement in 3-year disease-free survival, with an absolute reduction of approximately 7% when chemotherapy was given before surgery compared to after surgery [5, 6]. Most importantly, patients had less chemotherapeutic toxicity and consequently increased compliance rates. Furthermore, there is also interest in organ preservation after neoadjuvant therapy in those with clinically complete response, defined as no evidence of rectal cancer on digital rectal examination, endoscopic assessment, and MRI and/or FDG-PET/CT [7].

This has reignited interest in the use of [^18^F] Fluorodeoxyglucose- positron emission tomography /MRI (FDG-PET/MRI) as an effective imaging modality to improve accuracy of staging in colorectal cancer. It was first approved for clinical use back in 2011 in the United States of America and sought to combine the strengths of both PET and MRI modalities. It harnesses the strengths of MRI in defining anatomy, and offers diffusion- weighted imaging (DWI), spectroscopy, blood oxygenation level-dependent imaging in functional MRI, T1/T2 mapping and dynamic contrast-enhanced imaging. Contrast-enhanced MRI is an established imaging modality that has numerous clinical applications due to its superb soft tissue contrast and lack of ionizing radiation, and the ability to assess cellular density by diffusion-weighted imaging (DWI) and tissue perfusion by dynamic contrast-enhanced (DCE) [6, 8, 9]. Importantly, this has the potential to complement the metabolic imaging provided by FDG-PET. This assessment of metabolic activity supplements size/ morphology information and developed protocols have shortened scanning time and allowed correction of movement, hence improving the appeal of this imaging modality as an adjunct to colorectal cancer care [10].

Certainly, different protocols have been developed to streamline the acquisition process of PET/MRI—to save patient/scanner time while optimizing the PET/MRI sequence. The focus has been on timing MRI sequence to better match each PET bed position without compromising diagnostic quality so the images are acquired simultaneously. This process commences with a localizer, comparable to the topogram in a PET/CT scan, to be used for planning both the PET bed positions and the MRI sequences at each bed position. Thereafter, a specific MRI attenuation correction sequence is obtained at each bed position to create the attenuation correction maps for PET reconstruction. Finally, depending on the region being imaged and the pathology of interest, several other sequences have been developed to be performed at each bed position. This allows the complementary advantages of FDG-PET and MRI to theoretically improve our diagnostic accuracy and treatment decision-making for colorectal cancer [11].

It is also worth noting that FDG-PET/MRI does involve radiation exposure compared to MRI and PET/MRI has a significantly lower radiation dose compared to PET/CT, which is particularly beneficial in younger patients [12]. Therefore, the combination of FDG-PET and MRI in an integrated (hybrid) FDG-PET/MRI system promises to have a positive impact on disease diagnosis, staging, and restaging [13].

Its high sensitivity and its efficacy have been reported in the accurate localization of various urological and gynecological cancers [14, 15] but a wide consensus on the clinical usefulness of FDG-PET/MRI in the staging and restaging of colorectal cancer patients has yet to be reached. This systematic review aims to evaluate the effectiveness, summarizing the existing literature and discuss the emerging role of hybrid FDG-PET/MRI in colorectal malignancies which is currently underutilized.

## Methods

### Search strategy

A computer-assisted search of four databases (PubMed, EMBASE, Medline) were conducted for articles published between the years of 2000 and 2023. The following medical subject heading (MeSH) terms and text words were used for the search in all possible combinations: “PET/MRI” AND “colorectal cancer” OR “colorectal cancer imaging”. The cited references in each retrieved paper were also checked to ensure that all publications relevant to this study were captured. The last search date for this study was 30 June 2023.

### Selection of studies

This study was conducted in accordance to the Preferred Reporting Items for Systematic Reviews and Meta-analyses (PRISMA) guidelines [16]. Two reviewers (H.L. and K.C.) screened all article titles and abstracts, with all potentially relevant studies then subsequently selected for full text review. The selection of articles was based on the following inclusion criteria: adult population, all prospective or retrospective studies reporting on the performance of FDG-PET/MRI—accuracy in initial staging, restaging, and detection of metastatic disease, in addition to comparison with Standard of Care imaging (SCI) in colorectal cancer care. Studies that did not report on the performance of FDG-PET/MRI and SCI were excluded. All non-English studies, letters and conference abstracts were also excluded.

### Data extraction

Two reviewers independently extracted the data from the included studies using a standard data extraction form based on the PRISMA guidelines. Extracted information included those related to study information: country, study type, time period; study population and participant demographics: sample size, age, gender, imaging modalities utilized; indication of imaging; sensitivity and specificity of imaging modalities utilized; positive and negative predictive values of imaging modalities utilized and changes in management. Any discrepancies were resolved by consensus between the two reviewers and the supervising author (J.K.).

## Results

### Study characteristics

**Fig. 1:**
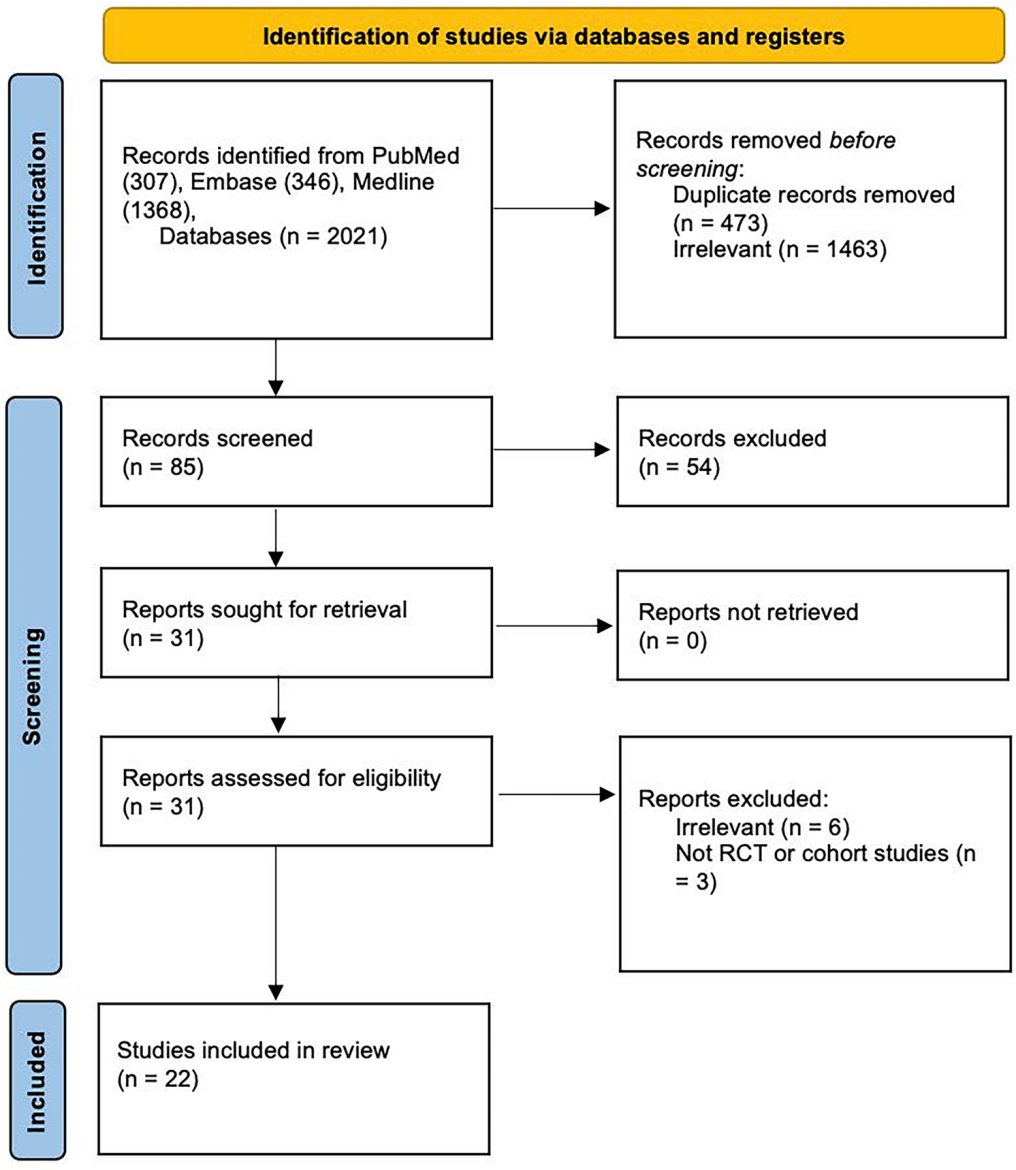
PRISMA flow diagram

Our search identified 85 non-duplicate and relevant studies from electronic databases. After screening titles and abstracts, 31 were identified for full-text reading and 22 of these articles were eventually included in our study cohort (Fig. 1) [11, 13, 17–36].

### Methodological quality

All 23 studies were assessed with the Quality Assessment of Diagnostic Accuracy Studies (QUADAS-2) tool using four domains—patient selection, index test, reference standard and flow and timing. The risk of bias for patient selection was high in many papers. Correspondingly, the flow and timing were also high.

### Clinical research design

There were ten prospective studies,  eleven retrospective studies and one pilot study (Table 1). This accounted for a total of 988 patients, with 582 rectal cancer patients. Disease staging was the most common indication for the scan, either on initial diagnosis or after neoadjuvant Chemoradiotherapy (nCRT) in rectal cancer.

**Table 1:** Demographics of all included studies

Year	Author	Study type	Number of pt	Age	Indication	Sen PET/MRI	Sen other	Spec PET/MRI	Spec other	PPV PET/MRI	PPV other	NPV PET/MRI	NPV other	Change in management	Upstaging	Upstage discordant findings	Downstaging	Downstage findings	Overall PET/MRI accuracy	Overall other accuracy
**2022**	Quieroz	Prospective	101	62.0	Staging	NR	NR	NR	NR	NR	NR	NR	NR	NR	NR	NR	NR	NR	NR	NR
**2021**	Catalano	Retrospective	62	54.0	Baseline staging	NR	NR	NR	NR	NR	NR	NR	NR	NR	NR	NR	NR	NR	NR	NR
**2021**	Furtado	Retrospective	31	53.0	Staging + restaging post neoadjuvant treatment	NR	NR	NR	NR	NR	NR	NR	NR	19%	19.35%	Peritoneal nodule, LN, local invasion of pelvic floor, differentiating post treatment change	NR	NR	90%	71%
**2021**	Plodeck	Retrospective	44	60.0	Follow-up	94%	88%	88%	75%	97%	94%	78%	60%	NR	NR	NR	NR	NR	93%	85%
2021	Quieroz	Prospective	101	62.0	Staging	90.80%	98.50%	86.10%	66.70%	NR	NR	NR	NR	NR	12.90%	Upstaged M0 to M1	NR	NR	88%	83%
2021	Zhou	Prospective	56	59 (median)	Staging/restaging	NR	NR	NR	NR	NR	NR	NR	NR	7.10%	10.70%	Operative to non-operative	7.10%	Non-operative to operative	99%	48%
2021	Zhang	Prospective	24	60.0	Gross tumor volume	NR	NR	NR	NR	NR	NR	NR	NR	NR	NR	NR	NR	NR	NR	NR
**2020**	Catalano	Retrospective	26	61.2	14 were diagnostic, 12 were restaging	NR	NR	NR	NR	NR	NR	NR	NR	30.77%	15.38%	Regional/non regional LN + peritoneal deposits	11.54%	Prostatic inflammation, reactive lymphadenopathy, post-surgical fistula	NR	NR
**2020**	Crimi	Prospective	36	68.5	Restaging post neoadjuvant treatment	T: 100% vs 96% N: 92% vs 86%	NR	T: 73% vs 73%; N: 92% vs 92%	NR	T: 89% each; N: 82 vs 78%	NR	T: 100% vs 96%; N: 96% vs 89%	NR	11%	11.11%	T0 to T1 require LE; regional LN	0	0	T: 92%; N: 92%	T: 89%; N: 86%
**2020**	Li	Retrospective	34	58.0	Staging/restaging	N + : 68.20% M: 100%	N + : 72.70% M: 93.80%	N + : 87.50% M: 84.80%	N + : 79.20% M: 89.20%	NR	NR	NR	NR	NR	NR	NR	NR	NR	M: 89.80%	M: 97.50%
**2020**	Yoon	Prospective	71	61.0	Metastatic work up	94%	94%	98%	73%	NR	NR	NR	NR	NR	NR	NR	NR	NR	NR	NR
**2019**	Amorim	Retrospective	39	56.7	Restaging/surveillance	NR	NR	NR	NR	NR	NR	NR	NR	35.70%	21.50%	NR	14.20%	NR	NR	NR
**2019**	Ferri	Prospective	30	62.2	Restaging post neoadjuvant treatment	91%	NR	85%	NR	95%		75%	NR	NR	NR	NR	NR	NR	90% (73% - 99%)	-
**2019**	Plodeck	Retrospective	44	60.0	Follow-up	94%	NR	94%	NR	97%	NR	90%	NR	NR	NR	NR	NR	NR	NR	NR
**2019**	Rutegards	Prospective	24	71.0 (median)	Staging	NR	NR	NR	NR	NR	NR	NR	NR	0%	8.30%	Upstaged to M1 disease	NR	NR	NR	NR
**2016**	Brendle	Retrospective	15	45.0	Metastatic work up	59%	58%	87%	94%	NR	NR	NR	NR	NR	NR		NR	NR	69%	66%
**2016**	Kang	Retrospective	51	60.2	Post-treatment follow-up and initial staging	91.80%	80.50%	92.60%	90.50%	NR	NR	NR	NR	21.60%	54.55%	Liver metastasis/ local recurrence	45.45%	Disproving local recurrence/metastatic analysis	NR	NR
**2016**	Lee	Retrospective	55	63.0	NR	NR	NR	NR	NR	NR	NR	NR	NR	NR	NR	NR	NR	NR	NR	NR
**2015**	Lee	Prospective	59	58.3	Staging + restaging	T: 92.90%	NR	NR	NR	NR	NR	NR	NR	NR	NR	NR	NR	NR	T: 92%	NR
**2015**	Papsulati	Pilot	12	59.0 (median)	Staging/restaging	86%	71%	100%	100%	NR	NR	NR	NR	NR	NR	NR	NR	NR	NR	NR
**2014**	Al-Nabhani	Prospective	50	47.2	Staging	NR	NR	NR	NR	NR	NR	NR	NR	10%	2%	Upstaged disease from T3A to T4, two additional lesions identified	4%	Down staged disease from T3 to T2, down staged disease from T4 to T3	NR	NR
**2009**	Kam	Retrospective	23	60.0	Staging pre-treatment	44% (for LN studies)	NR	100%	NR	100%	NR	NR	NR	0%	0%	NR	0	NR	NR	NR

*Pt* patient, *sen* sensitivity, *spec* specificity, *PPV* positive predictive value, *NPV* negative predictive value, *LN* lymph nodes, *T* tumor, *N* nodal, *M* metastasis

Indeed in 22 studies, the main endpoint was the ability of FDG-PET/MRI to stage colorectal cancer, be it primary or local recurrent disease. A comparison was drawn between FDG-PET/CT and FDG-PET/MRI performed in the same populations in ten studies. A comparison was drawn between FDG-PET/MRI and MRI performed in the same populations in seven studies.

## PET/MRI for initial staging

In this present review, 22 studies assessed FDG-PET/MRI use in the staging setting for the purpose of detecting burden of primary disease.

### Primary tumor

Integrated FDG-PET/MRI proved to be of greater diagnostic value. While the high soft tissue resolution provided by MRI allows for reliable assessment of T stage and the circumferential resection margin, the combination of FDG-PET with MRI can help in both lesion detection as well as margin delineation of the primary tumor planning beyond the muscularis propria [17, 18, 25].

### Nodal disease

Compared to MRI alone, FDG-PET/MRI N-staging accuracy was once again superior: 78.3% to 79% for FDG-PET/MRI compared to 58% to 76.1% for MRI [17, 23]. This represents the greatest advantage of FDG-PET/MRI as one of the central issues in current rectal cancer management is the accurate characterization of small lymph nodes.

### Colorectal cancer

There were 8 studies analyzing the accuracy of staging for colorectal cancer [13, 20, 25, 29, 30, 32, 34, 36] (Table 2). Overall, the detection of metastatic disease by FDG-PET/MRI was higher at 69% to 99.5% compared to 47.5% to 71% for other imaging techniques like FDG-PET/CT, CT or MRI alone [13, 20, 29].

**Table 2:** Accuracy of FDG-PET/MRI in colon cancer

Year	Author	Number of pt	Age	Sen PET/MRI	Sen other	Spec PET/MRI	Spec other	PPV PET/MRI	PPV other	NPV PET/MRI	NPV other	Accuracy PET/MRI	Accuracy other
2021	Furtado	31	53.0	NR	NR	NR	NR	NR	NR	NR	NR	90% (5%)	71% (8%)
2021	Zhou	56	59.0 (median)	NR	NR	NR	NR	NR	NR	NR	NR	99.50%	47.50%
2020	Catalano	26	61.2	NR	NR	NR	NR	NR	NR	NR	NR	NR	NR
2019	Amorim	39	56.7	NR	NR	NR	NR	NR	NR	NR	NR	NR	NR
2016	Brendle	15	45.0	59%	58%	87%	94%	NR	NR	NR	NR	69%	66% (PET/CT)
2016	Kang	51	60.2	91.80%	80.50%	92.60%	90.50%	NR	NR	NR	NR	NR	NR
2015	Lee	59	58.3	94%	94%	98%	73%	NR	NR	NR	NR	NR	NR
2014	Al-Nabhani	50	47.2	NR	NR	NR	NR	NR	NR	NR	NR	NR	NR

*Pt* patient, *sen* sensitivity, *spec* specificity, *PPV* positive predictive value, *NPV* negative predictive value

### Rectal cancer

There were 12 studies analyzing the accuracy of staging for rectal cancer (Table 3) [11, 17–19, 22–24, 26–28, 33, 35]. Overall, FDG-PET/MRI accuracy was superior at 88.4% to 93% compared to 82.6% to 85% for conventional imaging modalities MRI and CT. When comparing FDG-PET/MRI with CT for Tumor (T) and Node (N) staging, FDG-PET/MRI had higher accuracy in both aspects (92%, 92% vs 89%, 86%).

**Table 3:** Accuracy of FDG-PET/MRI in rectal cancer

Year	Author	Study Type	Number of pt	Age	Sen PET/MRI	Sen other	Spec PET/MRI	Spec other	PPV PET/MRI	PPV other	NPV PET/MRI	NPV other	Accuracy PET/MRI	Accuracy other
2022	Quieroz	Prospective	101	62.0	NR	NR	NR	NR	NR	NR	NR	NR	NR	NR
2021	Catalano	Retrospective	62	54.0	NR	NR	NR	NR	NR	NR	NR	NR	NR	NR
2021	Plodeck	Retrospective	44	60.0	94%	88%	88%	75%	97%	94%	78%	60%	93%	85%
2021	Quieroz	Prospective	101	62.0	90.80%	98.50%	86.10%	66.70%	NR	NR	NR	NR	88.40%	82.60%
2020	Crimi	Prospective	36	68.5	T: 100% vs 96% N: 92% vs 86%	NR	T: 73% vs 73%; N: 92% vs 92%	NR	T: 89% each; N: 82% vs 78%	NR	T: 100% vs 96%; N: 96% vs 89%	NR	T: 92%, N: 92%	T: 89%, N: 86%
2020	Li	Retrospective	34	58.0	N + : 68.20% M: 100%	N + : 72.70% M: 93.80%	N + : 87.50% M: 84.80%	N + : 79.20% M: 89.20%	NR	NR	NR	NR	M: 89.80%	M: 97.50%
2020	Yoon	Prospective	71	61.0	94%	94%	98%	73%	NR	NR	NR	NR	NR	NR
2019	Ferri	Prospective	30	62.2	91%	NR	85%	NR	95%	NR	75%	NR	90% (73%-99%)	NR
2019	Plodeck	Retrospective	44	60.0	94%	NR	94%	NR	97%	NR	90%	NR	NR	NR
2019	Rutegards	Prospective	24	71.0 (median)	NR	NR	NR	NR	NR	NR	NR	NR	NR	NR
2015	Papsulati	Pilot	12	59.0 (median)	86%	71%	100%	100%	NR	NR	NR	NR	NR	NR
2009	Kam	Retrospective	23	60.0	44% (for LN studies)	NR	100%	NR	100%	NR	NR	NR	NR	NR

*Pt* patient, *sen* sensitivity, *spec* specificity, *PPV* positive predictive value, *NPV* negative predictive value, *LN* lymph nodes, *T* tumor, *N* nodal, *M* metastasis

### PET/MRI vs PET/CT

FDG-PET/MRI and FDG-PET/CT were directly compared in 10 papers, all focused on systemic staging in the detection of distant metastasis (Table 4) [13, 18, 20, 25, 28, 29, 33–36]. The sensitivity of FDG-PET/MRI was higher than that of FDG-PET/CT, ranging from 59 to 94% while FDG-PET/CT ranges from 58 to 88%. The specificity of FDG-PET/MRI ranges from 87 to 100% while FDG-PET/CT ranges from 75 to 100% [18, 29, 33].

**Table 4:** Comparison of FDG-PET/MRI vs FDG-PET/CT

Year	Author	No of pt	Age	Comparison	Sen PET/MRI	Sen other	Spec PET/MRI	Spec other	Change in management	Upstaging	Upstage discordant findings	Downstage	Downstage findings
2021	Furtado	31	53.0	SCI: CT/MRI/PET-CT *scans were < 4 weeks apart	NR	NR	NR	NR	19%	19.35%	Peritoneal nodule, LN, local invasion of pelvic floor, differentiating post treatment change	NR	NR
2021	Plodeck	44	60.0	18F-fluorodeoxyglucose-PET/MRI and MRI	94%	88%	88%	75%	NR	NR	NR	NR	NR
2021	Zhou	56	59.0 (median)	PET/MRI abdo-PET/CT F-FDG	NR	NR	NR	NR	7.10%	10.70%	Operative to non-operative	7.10%	Non-operative to operative
2020	Catalano	26	61.2	PET-CT vs PET/MRI	NR	NR	NR	NR	8/26	15.38%	Regular/non regional LN + peritoneal deposits	3/26	Prostatic inflammation, reactive lymphadenopathy, post-surgical fistula
2019	Amorim	39	56.7	CT/PET-CT/MR pelvis	NR	NR	NR	NR	35.70%	21.50%	NR	14.20%	NR
2019	Rutegards	24	71.0 (median)	FDG-PET/CT and FDG-PET/MRI	NR	NR	NR	NR	0%	8.30%	Upstaged to M1 disease	NR	NR
2016	Brendle	15	45.0	MRI/MRI-DWI/MRI PET/PET/CT (will use PET/MRI vs PET/CT)	59%	58%	87%	94%	NR	NR	NR	NR	NR
2015	Papsulati	12	59.0 (median)	Hybrid FDG PET/MRI-PET/CT	86%	71%	100%	100%	NR	NR	NR	NR	NR
2014	Al-Nabhani	50	47.2	CT/PET-CT/MR	NR	NR	NR	NR	10%	2%	Upstaged disease from T3A to T4, Two additional lesions identified	4%	Down staged disease from T3 to T2, Down staged disease from T4 to T3
2009	Kam	23	60.0	SCI: CT/MRI/PET-CT	44% (for LN studies)	NR	100%	NR	0%	0%	NR	0%	NR

*Pt* patient, *sen* sensitivity, *spec* specificity, *LN* lymph nodes, *M* metastasis

### PET/MRI vs MRI

FDG-PET/MRI and MRI were compared in 7 papers (Table 5) [11, 13, 17, 18, 23, 29, 35]. Three utilized FDG-PET/MRI to assess T and N stage while the remaining four assessed their performance in detecting metastatic disease. Combined, the sensitivity of combined FDG-PET/MRI was comparable to that of MRI, ranging from 59% to 90.8% versus 58% to 98.5% respectively with slightly superior specificity: 86.1% to 87% compared to 66.7% to 94% [11, 18, 29]. The addition of metabolic imaging in FDG-PET/MRI proved superior in detecting distant metastatic disease with significantly higher sensitivity for M staging (100% vs 93.8%). While showing slightly lower sensitivity for N staging (72.7% vs 68.2%), FDG-PET/MRI provided higher specificity for N staging (87.5% vs 79.2%) while MRI provided higher specificity for M staging (89.2% vs 84.8%).

**Table 5:** Comparison of FDG-PET/MRI vs MRI

Year	Author	No of pt	Age	Indication	Sen PET/MRI	Sen other	Spec PET/MRI	Spec other	Change in management	Upstaging	Upstage discordant findings	Downstaging	Downstage findings
2021	Catalano	62	54.0	T and N	NR	NR	NR	NR	NR	NR	NR	NR	NR
2021	Furtado	31	53.0	Distant Metastasis	NR	NR	NR	NR	19%	19.35%	Peritoneal nodule, LN, local invasion of pelvic floor, differentiating post treatment change	NR	NR
2021	Plodeck	44	60.0	Local recurrence	94%	88%	88%	75%	NR	NR	NR	NR	NR
2021	Quieroz	101	62.0	Distant Metastasis	90.80%	98.50%	86.10%	66.70%	NR	12.90%	Upstaged M0 to M1	NR	NR
2020	Li	34	58.0	N stage	N + : 68.20% M: 100%	N + : 72.70% M: 93.80%	N + : 87.50% M: 84.80%	N + : 79.20% M: 89.20%	NR	NR	NR	NR	NR
2016	Brendle	15	45.0	Distant metastasis	59%	58%	87%	94%	NR	NR	NR	NR	NR
2009	Kam	23	60.0	N stage	44% (for LN studies)	NR	100%	NR	0%	0%	NR	0%	NR

*Pt* patient, *sen* sensitivity, *spec* specificity, *LN* lymph nodes, *T* tumor, *N* nodal, *M* metastasis

### Change in management from using PET/MRI

Importantly, this translated to changes in management after FDG-PET/MRI identified disease not picked up previously. Nine papers reported changes in management that ranged from 7.1% to 21.6%, with a majority reporting upstaging which resulted in further surgeries or abortion of surgeries for chemotherapy instead [11, 13, 20, 22, 25, 28, 30, 34, 36]. This was based on pathological confirmation of nodal and distant disease and while the overall rates of false positives or false negatives were not reported, this deserves greater attention.

## Discussion

Our updated systematic review on FDG-PET/MRI use in colorectal cancer care has highlighted the potential for it to change management in up to 7.1% to 21.6% of cases. This significant advantage of FDG-PET/MRI with regards to lesion detection and characterization (T stage) as well as in quantifying N and M staging suggest that FDG-PET/MRI is an emerging modality that may prove useful in the management of colorectal cancer patients, supporting the move towards hybrid imaging modalities which have proven to be advantageous in other cancers.

Specifically, this change in management can be attributed to the higher soft tissue contrast from FDG-PET/MRI which confers higher sensitivity and accuracy in detecting involved lymph nodes. N staging requires more than anatomic imaging as metastatic lymph nodes can have similar appearance and size as a normal non-metastatic lymph node. Hence, FDG-PET is imperative for such diagnostic purposes because the standardized uptake values (SUV) of the metastatic lymph nodes are higher than normal lymph nodes [9, 37]. At the same time, combining FDG-PET with MRI can help better characterize small pelvic nodes < 1 cm, overcoming the limitation of PET/CT in borderline enlarged lymph nodes between 5 and 10 mm [38, 39]. In particular, the longer FDG-PET acquisition that is simultaneous to the MRI sequences of the pelvis appears to result in a higher sensitivity for small perirectal nodes, particularly those that measure less than 5 mm [40]. Together, this has contributed to the significant percentages of change in management as shown in our review. The majority of patients with change in management had been reported as upstaging, resulting in further surgeries or abortion of surgeries for chemotherapy instead. This shows the potential of FDG-PET/MRI in detecting advanced diseases that is not surgically indicated, allowing patients to avoid unnecessary surgical interventions.

In the assessment of the M stage, our study determined a clear superiority of FDG PET/MRI in comparison to CT both in the staging and detection of metastatic disease. Currently, MRI has established itself as the reference imaging technique for metastasis, particularly in colorectal liver metastases [41]. With PET/MRI, it further allows for increased accuracy in detecting hepatic masses in colorectal cancer by combining the multiphase MRI of the liver together with PET which can potentially provide increased sensitivity and specificity [20, 21]. This is particularly useful when assessing liver lesions that would have otherwise been too small to characterize or remained indeterminate on CT [41, 42]. To mitigate the known intrinsic weakness of MRI in identifying the metastatic lung lesions, new, specific sequences have recently been developed to address this issue. The use of ultra-short echo times (UTE) sequences has provided superior visualization of lung parenchyma, achieving greater sensitivity in the detection of lung lesions [43, 44]. In a study done in 2022, UTE lung MRI showed close to 100% detection rate of nodules that were 5 mm or more in size but lower detection rate for nodules that were less than 5 mm in size. High resolution CT would then be more preferable in detecting metastasis in cancer patients but transition to UTE lung MRI should be done when following-up due to ionizing radiation exposure [45].

With the emerging paradigm shift in rectal cancer treatment with total neoadjuvant therapy (TNT) and watch/wait strategies proving non-inferiority in patients with clinical complete response (cCR), the accurate selection of patients with cCR has never been more crucial. Historically, FDG-PET/CT has shown sensitivity and specificity of 71% and 76%, respectively in predicting a histopathologically complete response after nCRT for rectal cancer [46]. It has been suggested that FDG-PET/MRI, by combining the already-reported good accuracy of PET images with the anatomical detail of MRI, could potentially improve on that [9, 38].

At present, the partial or complete replacement of viable tumor tissue with fibrosis has led to under-staging in some cases due to the inability of FDG-PET/CT to detect small clusters of residual disease or failure of morphological sequences (such as conventional T2-weighted MRI) and functional ones (such as diffusion-weighted imaging) to identify small remnant tumor deposits in the initial tumor site. By combining morphological, functional, and metabolic data, FDG-PET/MRI would be able to overcome these shortfalls and more accurately re-stage patients after nCRT [46, 47]. The integration of radiomics in an artificial intelligent model promises to enhance this capability. Recently, Giannini et al. compared 52 patients with locally advanced rectal cancer and how their histology results corresponded to texture features from FDG-PET/CT and MRI images [48]. Building on this, other studies have combined FDG-PET and MRI radiomics features together and showed greater sensitivity/specificity over models including only MRI features [42].

Equally, the authors acknowledge the inherent limitations of FDG-PET/MRI technology. While the main drawback of long acquisition time averaging 30 min, as well as the high costs have been widely reported, there are still several technical considerations required in the design and operation of the imaging systems. Specifically, modifications to conventional imaging systems that accommodate integration of the two modalities without image-degrading cross talk require deeper understanding before the technology can be widely adopted. Furthermore, there would be technical difficulties involved as PET detectors and not compatible with magnetic fields that will be present in MRI [49]. The integration of FDG-PET/MRI affords the opportunity for enhanced protocols and clinical applications which are currently being explored to overcome this [9].

In FDG-PET imaging, the limitations of standardized uptake value (SUV) cutoff, commonly used as a semi-quantitative value that measures tissue radioactivity concentration hence determining positive disease, could be overcome by volume-based FDG-PET/MRI parameters such as metabolic tumor volume (MTV) and total lesion glycolysis (TLG) to measure metabolic activity in the entire tumor mass. Our review confirmed the widely accepted SUV cutoff between benign and malignant lesions with a range from 2.0 to 2.5. While percentage change in tumor SUVmax and changes in DWI post-neoadjuvant therapy are predictors of outcome, it may not reflect the heterogeneous nature of the tumor. MTV represents the dual characteristics of tumor volume and extent of FDG uptake by tumor tissues. TLG has been proposed as a more accurate parameter as it considers SUVmean and MTV [50]. With the smaller slices during longer PET acquisition phase in PET/MRI, it is hoped this will decrease the partial volume artifact with future studies eagerly awaited. These changes in metabolism seen on FDG-PET could complement the information gathered on MRI alone.

Our analysis also showed that the specificity of FDG-PET/MRI is lower than MRI in the detection of metastatic disease. However, FDG-PET/MRI has a large potential in this field considering its ability to pick up metastatic disease that was previously not picked up with SCI, leading to fewer unnecessary surgeries. Future larger studies should research deeper on the use of FDG-PET/MRI and its potential for management changes.

Finally, we acknowledge the scarce number of studies published on this topic and the low level of evidence for most of the included studies. These studies have often included colon and rectal cancer patients together. However, the important suggestions of its role in changing management call for a Randomized Control Trial (RCT)—with FDG/PET-MRI in one arm and standard care imaging in the other to determine the overall impact on patient outcome in terms of survival benefit for example.

## Conclusion

This combination of superior soft-tissue resolution of MRI with the picomolar sensitivity of FDG-PET brings great promise. Our findings support the importance of FDG-PET/MRI in the diagnosis and management of colorectal cancer, where FDG-PET/MR altered clinical management in up to 21% of cases. Further RCTs should consider the above in evaluating long term benefits/costs of this modality while developing protocols for its standardized use.

As we march towards an era of precision medicine in which the profiling of tumor types guides progressively more specifically targeted cytotoxic and biologic agents, it has driven demand for more accurate imaging modalities. While Artificial Intelligence (AI), radiomics and radio-genomics might present helpful adjuncts, the introduction of FDG-PET/MRI to the current clinical management of colorectal malignancies presents another pathway for a patient-centered treatment approach [13].

## Data Availability

The data that support the findings of this study are available on request from the corresponding author, H.L. The list of included articles have been included in the reference list.
